# Nurses’ perceptions, acceptance, and use of a novel in-room pediatric ICU technology: testing an expanded technology acceptance model

**DOI:** 10.1186/s12911-016-0388-y

**Published:** 2016-11-15

**Authors:** Richard J. Holden, Onur Asan, Erica M. Wozniak, Kathryn E. Flynn, Matthew C. Scanlon

**Affiliations:** 1 Department of BioHealth Informatics, Indiana University School of Informatics and Computing, Indianapolis, IN USA; 2 Center for Patient Care and Outcomes Research, Division of General Internal Medicine, Department of Medicine, Medical College of Wisconsin, Milwaukee, WI 53226 USA; 3 Department of Pediatrics, Division of Critical Care, Medical College of Wisconsin, Milwaukee, WI USA

**Keywords:** Technology acceptance model, Pediatric intensive care, Nursing informatics, Usability, Human-computer interaction

## Abstract

**Background:**

The value of health information technology (IT) ultimately depends on end users accepting and appropriately using it for patient care. This study examined pediatric intensive care unit nurses’ perceptions, acceptance, and use of a novel health IT, the Large Customizable Interactive Monitor.

**Methods:**

An expanded technology acceptance model was tested by applying stepwise linear regression to data from a standardized survey of 167 nurses.

**Results:**

Nurses reported low-moderate ratings of the novel IT’s ease of use and low to very low ratings of usefulness, social influence, and training. Perceived ease of use, usefulness for patient/family involvement, and usefulness for care delivery were associated with system satisfaction (R^2^ = 70%). Perceived usefulness for care delivery and patient/family social influence were associated with intention to use the system (R^2^ = 65%). Satisfaction and intention were associated with actual system use (R^2^ = 51%).

**Conclusions:**

The findings have implications for research, design, implementation, and policies for nursing informatics, particularly novel nursing IT. Several changes are recommended to improve the design and implementation of the studied IT.

## Background

“Oh, people will come, Ray. People will most definitely come.” – A character in the film *Field of Dreams (1989)*, assures Iowa farmer Ray Kinsella if he builds a baseball diamond in his cornfield, fans will come to watch the game.

The *field of dreams fallacy* [1] applied to health information technology (IT) states it is not the case that “If you build IT, will they come (to use it)” [2]. Decades of research linking health IT to improved quality, efficiency, and patient safety are tempered by numerous findings that health IT’s intended end-users are at times dissatisfied with implemented IT, do not accept or use it, use a small portion of available features, work around it, and actively resist or even abandon it [3–8]. End-user perception, acceptance, and use of health IT have received increasing attention and are unavoidable in light of recent reports of provider dissatisfaction with aspects of electronic health record (EHR) systems [9–11]. While health IT has undoubtedly become more commonplace and increased in functionality, its value ultimately depends on end users perceiving it favorably, accepting it, and appropriately using it for patient care [12, 13].

Nurses’ perceptions, acceptance, and use of new health IT are particularly important because of: a) the variety of systems, including EHR, used by nurses [14] and b) nurses’ pivotal role in care delivery [15]. Thus, thought leaders and others in nursing informatics urge research on nurses’ acceptance and other implementation issues [16, 17]. However, relatively few studies assess nurses’ health IT perceptions or acceptance, as illustrated in various reviews [18, 19]. For example, Strudwick’s 2015 review of articles published 2000–2013 identified only 13 journal articles of this kind [20]. Notable examples are Carayon et al.’s [21] study of intensive care unit (ICU) nurses’ EHR perceptions and acceptance three and twelve months after EHR implementation; Holden et al.’s [22] modeling study of pediatric hospital nurses’ acceptance of bar-coded medication administration; Mailett et al.’s [23] acceptance modeling study of 616 nurses using electronic patient records in four Canadian hospitals; and Laerum et al.’s [24] study of use of, performance with, and satisfaction with a new electronic medical records system. Those studies all found variation in nurses’ IT acceptance and multiple predictors of acceptance, including the IT’s perceived usefulness and ease of use. Those and other studies of nurses’ IT acceptance urge continued research and the need to:

- Use established models of IT acceptance, such as the Technology Acceptance Model (TAM), as the foundation for nursing informatics research, design, and implementation;
- Extend existing models such as TAM to include additional variables such as social influence to use the system; and
- Contextualize existing models such as TAM to the unique case of nursing care, for example, operationalizing perceived usefulness of an IT as perceived usefulness for direct patient care [17, 20, 22, 23, 25–28].

In accordance with these recommendations, the present study applies an extended, contextualized TAM to examine pediatric ICU nurses’ perceptions, acceptance, and use of a novel health IT, the Large Customizable Interactive Monitor. This IT, henceforth shortened to Interactive Monitor, is an in-room, wall mounted screen displaying EHR data for clinician and patient/family use. To our knowledge, its acceptance and use have never before been studied. Our study took place in the first pediatric ICU in the US to implement the Interactive Monitor. It therefore represents an important step in assessing nurses’ response to a novel IT with potential benefits to patient care. From the perspective of technology acceptance modeling in the domain of health IT or nursing informatics, the study is novel in including variables specifically adapted to the study context and examining the relationship between acceptance and use.

## Methods

The study was a cross-sectional survey of pediatric ICU nurses. Data collection occurred in summer of 2015 and was approved by the Medical College of Wisconsin Institutional Review Board.

### Conceptual model

The study’s theoretical framework was adapted from TAM, [29] a paradigmatic behavioral theory of IT acceptance and the leading theory applied in health IT acceptance research [26, 30]. TAM posits IT perceptions lead to its acceptance and acceptance results in actual use. TAM research variably defines acceptance as satisfaction with an IT system or the intention to use it [31]. The two IT perceptions canonically associated with acceptance are IT ease of use and usefulness, but perceptions of social influence to use IT, facilitating conditions, and motivation have also been included as predictors of acceptance in the literature [32, 33]. Holden and colleagues have argued the classic TAM is not suitable for explaining contemporary health IT acceptance and note various revisions of TAM in the IT acceptance literature, [32–35] as well as inconsistencies between how TAM constructs are operationalized and the unique nature of healthcare [12, 22, 25, 26, 36–39]. In particular, they argue the following five points:

- Expanding the concept of perceived ease of use. Perceive ease of use of health IT involves more than low mental effort, as it is traditionally defined; ease of use also includes specific aspects of usability such as learnability and ease of navigation [12, 38]. For this study, we hypothesized an expanded measure of perceived ease of use will have good internal consistency and will be associated with IT acceptance (Hypothesis 1, H1).
- Contextualizing the concept of perceived usefulness. Perceived usefulness of health IT is more than its impact on productivity, as traditionally defined, and includes specific benefits for healthcare delivery such as improved safety, more effective patient care, or patient engagement [12, 22, 38]. For this study, we hypothesized that contextualized measures of perceived usefulness, related to patient care and patient engagement, would be associated with IT acceptance (Hypothesis 2a, H2a), but a traditional perceived usefulness measure would not (Hypothesis 2b, H2b).
- Adding the concept of social influence. Health IT acceptance and use behavior are shaped by internal and external social forces; clinicians experience social influence from colleagues, patients, organizational leaders, and entities outside the organization; perceived social influence should be included when studying acceptance of health IT [22, 36]. For this study, we hypothesized that measures of social influence, related to the institution and patients/families would be associated with IT acceptance (Hypothesis 3, H3).
- Adding the concept of barriers and facilitators. Health IT acceptance and use behavior are constrained or enabled by a variety of barriers or facilitators such as training and technical support; perceived barriers and facilitators should be included in models of health IT acceptance [22, 37]. For this study, we hypothesized that the facilitator of training on the system would be associated with IT acceptance (Hypothesis 4, H4).
- Examining satisfaction, intention to use, and the nature of health IT use. Health IT acceptance can be conceptualized as a combination of intention to use the health IT and satisfaction with the IT, as intention may result in baseline use, but satisfaction may influence the completeness of health IT use and potential workarounds [22, 25, 39]. For this study, we hypothesized that nurses’ beliefs would be associated with both satisfaction with IT and intention to use IT (Hypotheses 5a and 5b, H5a and H5b). Further, we hypothesized that satisfaction and intention to use would be associated with a measure of how completely the IT is used (Hypothesis 6, H6).

**Fig. 1:**
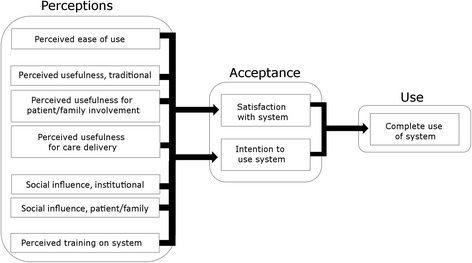
Study conceptual model, adapted from Technology Acceptance Model based on proposed extensions in Holden et al. [12, 22, 25, 26]

Accordingly, this study tests an adapted TAM, with constructs added based on newer versions of TAM and adapted to the healthcare context. Specifically, this study: 1) expanded traditional measures of perceived ease of use to include learnability and navigability; 2) supplemented traditional measures of perceived usefulness with variables of perceived usefulness for patient/family engagement and care delivery; 3) added measures of social influence from the institution and patients and families; 4) added a measure of perceived training on the system; and 5) measured intention, satisfaction, and completeness of use. Figure 1 shows the measured variables and hypothesized relationships.

### Setting

The study was performed in the 72-bed pediatric ICU of a freestanding children’s hospital in a mid-sized Midwestern city. The pediatric ICU had three floors with 24-beds each for cardiac, surgical, and medical ICU subunits.

### The interactive monitor

The hospital implemented a new EHR system in 2012 and at the same time became the third hospital—and the very first pediatric hospital—in the nation to install this IT using Epic Monitor technology (v 2010, Epic Systems Corporation, Verona, WI). This system was a 42” (diagonal) flat panel touch screen monitor displaying validated view-only patient information chosen by the hospital, including vital signs, laboratory results, medications, and interventions recorded in the EHR. Physiologic measures were only displayed if they were reviewed by a nurse, distinguishing the system from physiologic monitors. These systems were mounted in every patient room in the pediatric ICU, were accessible without repeated log-in, and were intended for use by clinicians and patients or their families. A novel aspect of the system was that the displayed information could be configured by the hospital and would be populated directly from the EHR. Any new data element in the EHR could therefore also be eventually displayed on the monitor. Another novel aspect was its interactive nature, namely, offering the ability to scroll, expand, or “drill down” to access additional content. More information on this system along with photographs are provided in Appendix A.

### Procedure

Every nurse in the unit, unless still in training, received a paper survey June-August, 2015. The goal for recruitment was to include as many of the nurses in the unit as possible, with a minimum sample size to perform our modeling analysis using a ratio of 10 participants for every model variable. The survey had 50 items about the system, of which 28 were used in the present analysis (Table 1 and Appendix B). Per Table 1, most survey items and scales were drawn from prior work, but some were newly created for the study to further explore technology acceptance model development. Each survey item used a 7-point intensity response scale with response categories as follows: 0 (not at all), 1 (a little), 2 (some), 3 (a moderate amount), 4 (pretty much), 5 (quite a lot), 6 (a great deal), and don’t know. The scale was the same one used in four prior or ongoing studies of health IT acceptance among nurses, pharmacy workers, primary care providers, and mental/behavioral health providers. In those studies, responses have been reported across all response categories, although lower responses have been more likely. The average standard deviation has been around 1.4-1.6 on the 7-point scale. Surveys were supplemented by 10 hours of unstructured observations of clinicians using the system and qualitative, and semi-structured in-person clinician interviews (*n* = 39). More detail on interview and observation data collection and analysis methods is available elsewhere [40]. Observation and interview findings are not formally reported here but contributed to our interpretation of the survey findings and recommendations for system redesign.

**Table 1:** Survey scales and items, their source, and internal consistencies. (For precise item wording, see Appendix B)

Scale and items	Source	Cronbach’s alpha
Perceived ease of use, expanded (6 items) • Clear and understandable • Easy to use • Requires a lot of mental effort • Easy to get it to do what I want • Easy to learn • Easy to navigate	TAM; Venkatesh & Morris [55] + two new items created based on usability definitions (learnability, navigability) [43]	0.873
Perceived usefulness, traditional (4 items) • Improves job performance • Increases productivity • Enhances effectiveness in job • Useful in job	TAM; Venkatesh & Morris [55]	0.929
Perceived usefulness for patient/family involvement, contextualized (4 items) • Improves patient/family interaction • Improves sharing information with family • Improves communication with family • Improves family engagement	Newly created for study, based on nursing TAM; Holden et al. [22]	0.941
Perceived usefulness for care delivery, contextualized (4 items) • Improves patient care • Improves information organization • Improves access to patient information • Improves sharing info with care team	Nursing TAM; Holden et al.,[22] and adapted to the study context	0.916
Social influence, institutional (3 items) • Institution thinks I should use it • Supervisors think I should use it • Colleagues think I should use it	Modified from TAM research; Venkatesh et al.,[56] based on normative IT use research; Holden [36]	0.891
Social influence, patient/family (1 item) • Patients/families like that I use it	Nursing TAM; Holden et al. [22]	n/a
Perceived training on system (2 items) • Received adequate training • Training was clear	Nursing TAM; Holden et al., [22] based on Bailey & Pearson, [57] and adapted to the study context	0.908
Satisfaction with system (2 items) • Satisfied with system • Would recommend it to others	Nursing TAM; Holden et al.[22]	0.883
Intention to use system (2 items) • Intend to use in next 6 months • Want to use it	TAM; Venkatesh & Morris [55]; 2 item version based on Holden et al. [22]	0.903
Complete use of system (2 items) • Use all available features • Skip/ignore parts (reverse scored)	Nursing TAM; Holden et al., [22] adapted to the study context	0.615

*TAM* technology acceptance model; optimal Cronbach’s alpha value is > 0.70 and higher values are indicative of internal consistencies; the response scale was 0 (not at all), 1 (a little), 2 (some), 3 (a moderate amount), 4 (pretty much), 5 (quite a lot), 6 (a great deal), and don’t know [22, 25]

### Analysis

Survey items with high item nonresponse or “don’t know” responses were eliminated, except in the case of the two social influence measures, for which “don’t know” and “not at all” responses were aggregated (under the assumption that not knowing of others’ expectations produces no social influence). Scale items were examined for missing data. The mean rate of missing items was 3% and rates did not differ much between perception scales (2-4%). There were slightly more missing items for the satisfaction (6%), intention (5%), and use (6%) scales. Scales were constructed according to Table 1 by averaging items with a floating denominator to address item nonresponse; thus the range of scale scores could be between 0 and 6. Internal consistencies among scale items were calculated using Cronbach’s alpha; Cronbach alpha values were good to excellent for all perception scales (all greater than 0.87) and acceptance scales (all greater than 0.88), but lower than optimal for the 2-item use scale (0.61).

The conceptual model in Fig. 1 was tested with separate models for satisfaction and intention using stepwise linear regression based on minimizing the Akaike information criterion (AIC). We also fitted regression models based on the same stepwise model selection process after aggregating the two contextualized perceived usefulness scales as well as the two social influence measures. All models resulting from automated variable selection processes were compared to full multiple regression models (i.e., no variable removal). Results were similar across all models; thus we report only the stepwise regression results of the disaggregated model (Fig. 1). Linear regression was also used to evaluate system use as an outcome with satisfaction and intention as predictors. Log and square root transformations of the outcomes did not substantially improve model fit, and so the untransformed results are presented. The R statistical package (R Foundation for Statistical Computing, Vienna, Austria) was used for analysis.

## Results

A total of 167 out of 230 eligible nurses adequately completed the survey, a response rate of 72.6%. Respondent characteristics are reported in Table 2a.

**Table 2:** (a) Respondent characteristics and descriptive statistics for (b) perceptions, (c) acceptance, and (d) use

(a) Respondent characteristics (*N* = 167)	Count (%)
Age	
18–29	73 (44.8)
30–39	52 (31.9)
40–49	19 (11.7)
50–59	15 (9.2)
60+	4 (2.5)
Gender	
Female	150 (91.5)
Race and ethnicity	
White/European American	157 (96.9)
Black/African American	2 (1.2)
Asian	1 (0.6)
American Indian/Alaska Native	1 (0.6)
No response	5 (3.0)
% Hispanic, of those responding	5 (3.1)
Years of experience with any EHR/current EHR	
0–1	9 (5.7)/31 (18.8)
1–2	19 (12.0)/30 (18.2)
2–3	77 (48.7)/104 (63.0)
> 3	53 (33.5)/0 (0.0)
Years at hospital	
Mean (SD)	8.9 (9.2)
(b) Perceptions (N = 167)	Mean (SD)
Perceived ease of use, expanded	3.88 (1.52)
Perceived usefulness, traditional	2.03 (1.71)
Perceived usefulness for patient/family involvement, contextualized	2.58 (1.81)
Perceived usefulness for care delivery, contextualized	2.05 (1.79)
Social influence, institutional	2.84 (1.70)
Social influence, patient/family	2.04 (1.91)
Training on system	1.06 (1.39)
(c) Acceptance (N = 167)	Mean (SD)
Satisfaction with system	2.16 (1.66)
Intention to use system	2.32 (1.62)
(d) Use (N = 167)	Mean (SD)
Complete use of system	1.89 (1.52)

*EHR* electronic health record system; The response scale for perceptions, acceptance, and use was 0 (not at all), 1 (a little), 2 (some), 3 (a moderate amount), 4 (pretty much), 5 (quite a lot), 6 (a great deal)

### Perceptions, acceptance, and use

Nurses’ perceptions of ease of use, usefulness, social influence, and training are reported in Table 2b. On average, respondents had moderate or higher ease of use ratings but low ratings of usefulness, particularly for patient care. Perceived institutional social influence to use the system were variable but low on average, and many nurses reported that patients and families had no opinion about nurses’ use of the system. Perceptions about training were particularly low, confirmed by our observations and interviews with nurses (unpublished) and other providers [40].

Acceptance, measured by satisfaction with and intention to use the Interactive Monitor, was also low (Table 2c). Nurses reported low satisfaction with and intention to use the system over the next six months.

Nurses’ self-reported use was also low (Table 2d). Nurses generally reported not using features of the system and skipping or ignoring parts of it.

### Testing the adapted model of technology acceptance

**Fig. 2:**
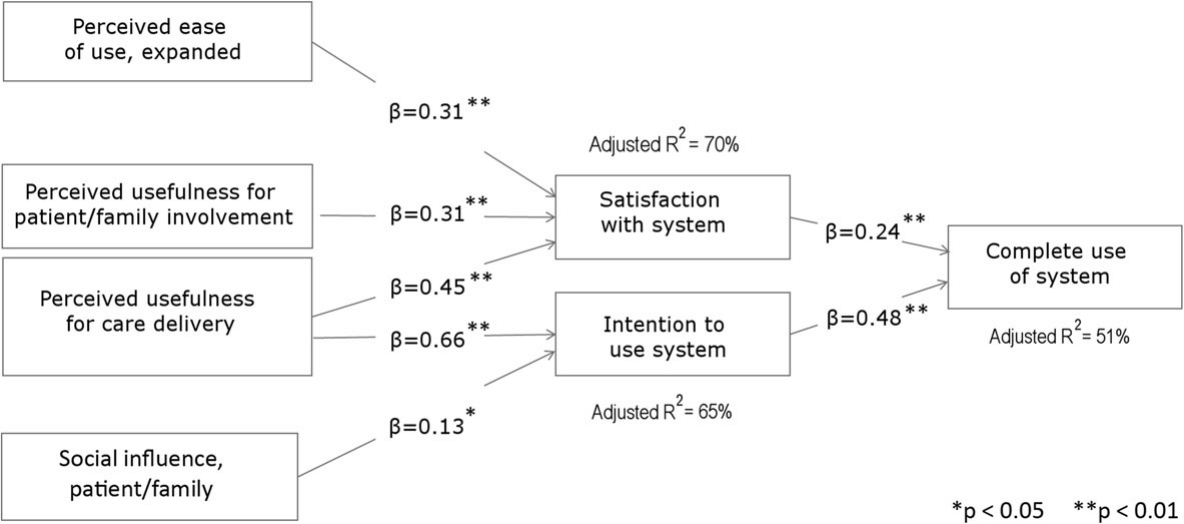
Stepwise regression results for the adapted model of technology acceptance. (Only retained model variables are shown)

Results of the stepwise regression test of the adapted TAM are depicted in Fig. 2 and fully detailed in Tables 3 and 4. For *satisfaction*, the perceptions retained in the final model were *perceived ease of use, expanded* (β = 0.31, *p* = 0.002, H1 supported), *perceived usefulness for patient/family involvement* (β = 0.31, *p* = 0.004, H2a supported), and *perceived usefulness for care delivery* (β = 0.45, *p* < 0.0001, H2a supported) (Table 3). These three perceptions explained 70% of the variance in satisfaction (model F(3,93) = 75.87, H5a supported). For *intention to use*, perceptions included in the model were *perceived usefulness for care delivery* (β = 0.66, *p* < 0.0001, H2a supported) and *patient/family social influence* (β = 0.13, *p* = 0.046, H3 supported) (Table 3). These two perceptions explained 65% of the variance in intention to use the system (model F(2,94) = 90.39, H5b supported). *Traditional perceived usefulness* (H2b supported) and *training perceptions* (H4 rejected) were not retained in either model.

**Table 3:** Stepwise linear regression results for the outcomes satisfaction and intention to use^a^

	Satisfaction		Intention	
	Estimate (SE)	*t*-value, *p*-value	Estimate (SE)	*t*-value, *p*-value
Intercept	−0.79 (0.41)	*t* = −1.90, *p* = 0.061	0.77 (0.22)	*t* = 3.54, *p* < 0.001
Perceived ease of use, expanded	0.31 (0.10)	*t* = 3.20, *p* = 0.002	^b^	
Perceived usefulness for patient/family involvement	0.31 (0.10)	*t* = 2.93, *p* = 0.004	^b^	
Perceived usefulness for care delivery	0.45 (0.10)	*t* = 4.41, *p* < 0.001	0.66 (0.07)	*t* = 9.13, *p* < 0.001
Social influence: Patients/Family	^b^		0.13 (0.06)	*t* = 2.02, *p* = 0.046
	Adjusted R^2^ = 0.70		Adjusted R^2^ = 0.65	

^a^Perceived usefulness, traditional; social influence, institutional; and perceived training on system were not significant in either model, and are not included in this table

^b^Not a statistically significant model covariate

**Table 4:** Stepwise linear regression results for the outcome complete system use

	Complete use of system	
	Estimate (SE)	*t*-value, *p*-value
Intercept	0.54 (0.20)	*t* = 2.71, *p* = 0.008
Satisfaction	0.24 (0.07)	*t* = 3.45, *p* < 0.001
Intention	0.48 (0.07)	*t* = 6.65, *p* < 0.001
	Adjusted R^2^ = 0.51	

Satisfaction and intention to use explained 51% of the variance in *self-reported actual use* (model F(2,154) = 83.57, H6 supported). The association for *satisfaction* (β = 0.24, *p* = 0.0007) was smaller than for *intention* (β = 0.48, *p* < 0.0001) (Table 4).

## Discussion

Based on present findings and those published elsewhere, we strongly refute the notion that implementing health IT results in actual use (i.e., the field of dreams fallacy [1]) and endorse the statement that “the benefits of healthcare technologies can only be attained if nurses accept and intend to fully use them” [20]. It is especially important to explore the perceptions of nurses toward novel technologies whose use is voluntary and investigate which perceptions correlate with acceptance and use. This is because, as we found, acceptance and use will vary. In the present study, these outcomes not only varied, but were on average quite low, putting in question the early returns on the hospital’s investment in the technology.

Moreover, when specific antecedents of acceptance and use are known, they can guide design, redesign, implementation strategies, and policies to promote appropriate acceptance and use [37, 38]. For example, we found that the Interactive Monitor was perceived as moderately easy to use and ease of use was associated with satisfaction, though not with intention to use. This suggests satisfaction, which correlates with actual use, could be improved through usability engineering and training, both of which nurses rated very poorly in this study. While early acceptance studies with physicians argued ease of use may not predict technology acceptance in healthcare, [41, 42] we have shown here and elsewhere the significance of ease of use for nurses’ satisfaction with health IT [22, 39]. Our perceived ease of use scale contained two items, learnability and navigability, not traditionally included in measures of the construct. These items are based on two key components of usability [43, 44] and we recommend their addition to future measures of perceived ease of use. Indeed, another recent study of nurses reported IT learnability as a high-priority system attribute [45].

The strongest predictors of acceptance were the two measures of perceived usefulness. Usefulness for patient care was the stronger of the two and was the only usefulness measure correlated with intention to use. This can be interpreted as nurses’ high concern for providing optimal patient care. Many nurses saw little or no value of the system, either for patient/family involvement or care delivery. In contrast, other studies have shown the objective performance usefulness of integrated visual displays for ICU nurses [46]. Our findings promote further attention to the usefulness of IT for care delivery in health IT acceptance. Further, our measure of social influence from patients and families—assessed as the degree to which nurses believed patients/families liked them using the system—was significantly, albeit weakly, associated with nurses’ intention to use the Interactive Monitor. These findings concerning patients and families are important because inpatients desire more involvement and technology [47] and have responded positively to large in-room information displays [48]. A recent review of inpatient technologies, including ones displaying patient-specific information, extolled the virtues of such systems for patient engagement [49]. However, to achieve actual value, nurses may need to agree about the system’s usefulness and actively facilitate patient and family use.

The findings validated our two novel, contextualized measures of perceived usefulness. Usefulness for patient/family involvement was newly created for the study and usefulness for care delivery was created in a prior study of nursing IT and adapted for this study. These new measures define usefulness based on both the hypothetical value of the Interactive Monitor and the meaning of “usefulness” in nursing care. Holden and colleagues have previously argued for conceptualizing usefulness this way, rather than the traditional TAM definition (as generally useful for workplace productivity); the latter measure was not correlated with acceptance in the present study when the contextualized measures were included.

Lastly, we note that social influence from the institution and perceptions of system training were not associated with nurses’ system acceptance, contrary to our hypotheses. A possible explanation for both is the restriction of range in nurses’ responses to these items, particularly regarding training. Nurses may also have weighed their personal, professional opinions of the system much more than the expectations of their supervisors, colleagues, and the institution. The influence of perceptions of training may also have been mediated by the conceptually related perceived ease of use and perceived usefulness.

As expected for a technology whose use was voluntary, [31] self-reported intention to use the studied IT was associated with actual use, and more strongly so than was satisfaction, the other measure of acceptance. Both acceptance measures were significantly associated with use, an important finding given that acceptance studies do not always assess actual use and in some cases find no correlation with acceptance [50]. Use in this study was conceptualized and measured in a novel manner: as *complete* use of the system, incorporating items on using all available features and skipping or ignoring parts of the system. Other technology acceptance researchers have argued for developing measures of use beyond “use/non-use,” including the completeness of system use [51, 52].

Having found low perceptions, satisfaction with, intention to use, and actual use of the Interactive Monitor, we suggest at the time of the study that this IT did not produce the results expected by the hospital or product vendor. A thorough exploration of the reasons for this is beyond the scope of this quantitative modeling study. However, based on observations and interviews, Table 5 provides several suggestions for improving the design and implementation of this technology toward achieving more favorable end-user perceptions, acceptance, and use.

**Table 5:** Recommendations for improving the Large Customizable Interactive Monitor, based on observations and interviews with nurses

• Incorporate whiteboard-like features: goals of the day, parent information (e.g., phone number, preferences), parents’ questions and concerns
• Add due dates or task lists for pending tasks (e.g., dressing change)
• Provide screen saver mode for glanceable information frequently accessed by families (e.g., photos of the medical team)
• Train nurses on the purpose of the Interactive Monitor, procedures for its use, recommendations for use, and basic information (e.g., origin of data in the system)
• Eliminate functions not useful for nurses
• Update the problem list more frequently
• Customize display to accommodate needs of nurses in the unit instead of generic information
• Consolidate flowsheet, drips, labs, and urine output, on single timeline
• Show interventions on a timeline to facilitate identification of intervention-related effects and trends
• Match fluids ins and outs to the timeframe used in medical records system
• Functionality showing the interventions that happened and how they affected the vital signs on a trended scale
• Incorporate a synopsis screen

### Study strengths and limitations

Study strengths included a focus on the sometimes neglected areas of nursing and pediatric health IT, [53] quantitative assessment of perceptions and acceptance, strong theoretical basis, and relatively large response rate. The study sample size was relatively large for health IT acceptance research and was greater than that of 63% of technology acceptance studies with nurses [20]. The use of standard construct definitions and measurements, as well as theory-driven expansions of these, was a strength and we urge others to reuse and build on these (see Appendix B for verbatim survey items). Limitations were studying a PICU at a single children’s hospital, the use of self-report to measure actual use, and the cross-sectional design. The scale measure of use had only two items and demonstrated lower than desirable internal consistency. The measure of social influence from patients/families was a single item and was worded as patient and families liking as opposed to *wanting* nurses’ use of the system. Further, additional variables could have been added to predict acceptance and use. The novelty of the technology and the very few hospitals implementing it precluded a multisite study. Although the survey was not designed to learn nurses’ reasons for system perceptions, we may speculate low perceived usefulness stemmed from the view-only nature of the system, meaning nurses could not enter or edit content through the system and did not directly control which content their unit displayed. Physicians’, nurses’, and families’ non-use of the system may have further reduced its usefulness. The novelty of the system, minimal training, and system lag may also have shaped nurses’ perceptions.

Future research is needed to address three methodological issues from this study. First, as new measures and concepts related to health IT acceptance are proposed and studied, a more rigorous assessment of the psychometric properties of individual items and scales will be necessary. This is somewhat limited by our recommendation that conceptualization and measurement be contextualized to the specific users, IT, tasks, and settings of use being studied. However, some conceptual and measurement standardization will be needed and this is demonstrated in the present study’s slight adaptation of prior research with nurses and pharmacy workers. Second, as measures are standardized and health IT acceptance models are solidified over multiple studies, analytic methods must shift from exploratory to confirmatory. Thus, for example, although the present study used stepwise linear regression, future work testing similar hypothesized relationships between health IT perceptions, acceptance, and use, could apply structural equation modeling or similar techniques. Third, this study’s conceptual model builds on TAM and subsequent iterations (TAM2, TAM3). In 2003, Venkatesh and colleagues combined TAM and other models to form the unified theory of acceptance and use of technology (UTAUT) [33]. Although criticized for being less parsimonious than TAM, UTAUT includes additional constructs and relationships which may help understand health IT acceptance and use. UTAUT has been fruitfully applied in the domain of health IT [54] but to be tested fully would require a larger sample size than the one in this study.

## Conclusions

Overall, this study appropriately contextualized a strong theory to measure pediatric ICU nurses’ perceptions, acceptance, and use of a novel voluntary health IT. It yielded important findings about the relationships between these constructs, lending insight into future design, implementation, and research on similar technologies. It also produced insights about measuring health IT perceptions, acceptance, and use. We encourage further theory-based examination of both in-room inpatient IT like the Large Customizable Interactive Monitor and other novel systems intended to improve care delivery and patient engagement.

## Appendix A

Additional description and illustration of the Large Customizable Interactive Monitor (LCIM).

## Appendix B

Survey items.

## Abbreviations

EHR: Electronic health record
ICU: Intensive care unit
IT: Information technology
TAM: Technology acceptance model
UTAUT: Unified theory of acceptance and use of technology
